# The adaptation of spike backpropagation delays in cortical neurons

**DOI:** 10.3389/fncel.2013.00192

**Published:** 2013-10-30

**Authors:** Yossi Buskila, John W. Morley, Jonathan Tapson, André van Schaik

**Affiliations:** ^1^Bioelectronics and Neuroscience Group, The MARCS Institute, University of Western SydneyPenrith, NSW, Australia; ^2^School of Medicine, University of Western SydneyPenrith, NSW, Australia

**Keywords:** action potential, somatosensory cortex, electrophysiology, cell attached, dendrite, adaptation, rat

## Abstract

We measured the action potential backpropagation delays in apical dendrites of layer V pyramidal neurons of the somatosensory cortex under different stimulation regimes that exclude synaptic involvement. These delays showed robust features and did not correlate to either transient change in the stimulus strength or low frequency stimulation of suprathreshold membrane oscillations. However, our results indicate that backpropagation delays correlate with high frequency (>10 Hz) stimulation of membrane oscillations, and that persistent suprathreshold sinusoidal stimulation injected directly into the soma results in an increase of the backpropagation delay, suggesting an intrinsic adaptation of the backpropagating action potential (bAP), which does not involve any synaptic modifications. Moreover, the calcium chelator BAPTA eliminated the alterations in the backpropagation delays, strengthening the hypothesis that increased calcium concentration in the dendrites modulates dendritic excitability and can impact the backpropagation velocity. These results emphasize the impact of dendritic excitability on bAP velocity along the dendritic tree, which affects the precision of the bAP arrival at the synapse during specific stimulus regimes, and is capable of shifting the extent and polarity of synaptic strength during suprathreshold synaptic processes such as spike time-dependent plasticity.

## INTRODUCTION

Neurons communicate with each other through the generation of action potentials, which are initiated in the axon initial segment and propagate to the axon terminal (orthodromic propagation), and back into the soma and dendrites (antidromic propagation or backpropagation; Spruston et al., 1995; Stuart et al., 1997b; Bernard and Johnston, 2003). The discovery of a multitude of voltage- and ligand-gated ion channels in the dendrites extended their original concept as passive tubes to include active complex computational properties (Stuart and Sakmann, 1995; Häusser and Mel, 2003; Waters et al., 2005; Froemke et al., 2010), establishing their role as integrative cellular elements. Previous reports showed that the propagation of the antidromic spike along the dendritic arbors depends on the type of neuron, and is highly dependent on Na^+^ conductance along the dendritic tree (Colbert et al., 1997; Stuart et al., 1997a) as well as dendritic morphology (Vetter et al., 2001). Other studies reported on the decremental fashion of the backpropagating action potential (bAP) invasion to the dendrites during a spike train and emphasized the contribution of A-type K^+^ currents in the attenuation of the bAP amplitude along the apical dendrite (Johnston and Hoffman, 1999; Bernard and Johnston, 2003), which could also be modified by the cholinergic agonist charbacol (Tsubokawa and Ross, 1997), suggesting network driven neuromodulation of the antidromic propagation. On the other hand, a recent *in vivo* study showed that bAP invasion to the distal dendrites is highly reliable across a wide range of brain states, network activity and stimulus conditions and is only mildly modulated by neuronal firing frequency, hence it is well suited to provide crucial signals for the control of synaptic plasticity (Bereshpolova et al., 2007).

In recent years, several studies have shown that the antidromic propagation of action potentials into the dendrites could significantly impact the way signals are processed in the central nervous system (Häusser and Mel, 2003; Nevian et al., 2007; Sjöström et al., 2008). More specifically it has been shown that spike backpropagation provides an associative link between the axonal output and the synapses, thus allowing them to follow a “Hebbian learning rule” such as spike time-dependent plasticity (STDP; Magee and Johnston, 1997; Dan and Poo, 2004; Nevian et al., 2007). In that regard, bAP in the dendrites has been demonstrated to be an important postsynaptic signal for the generation of long-term potentiation (LTP) in several brain regions such as hippocampus (Magee and Johnston, 1997) and neocortex (Markram et al., 1997; Stuart and Häusser, 2001; Sjöström et al., 2008). During backpropagation, the spikes cause depolarization along the dendrite and can interact with orthodromic signals arriving from the synapse to initiate calcium electrogenesis (Larkum, 1999). Therefore, the precise time of arrival of the bAP at the location of the synaptic input will determine the extent and polarity of any change in synaptic strength. Hence, the precision of the backpropagating spike is most important during situations in which the time window between the pre and postsynaptic signals is very short. In these cases, even slight alterations in the backpropagation delay, which are due to modification of the conduction velocity along the apical dendrite, will impact the delay time of the peak amplitude, which will result in modifications of the associated calcium electrogenesis and signal integration at the synapse, thus shifting the synaptic gain (Dan and Poo, 2006).

Since Hebbian synaptic plasticity acts as a positive feedback mechanism, it leads to destabilization of the neuronal network (Narayanan and Johnston, 2010). A recent simulation study argues that as a compensation for this instability, there is a modification of the synaptic threshold in an activity-dependent manner (Narayanan and Johnston, 2010), as proposed in the Bienenstock–Cooper–Munro (BCM)-like plasticity framework (Bienenstock et al., 1982), which is achieved through regulation of the *I*_h_ current in the dendrites. In this way, the threshold modulation acts as a negative feedback and returns the stability to the network. As an outcome of this regulation, it is argued that the membrane excitability will be modified. If so, this would affect the bAP propagation delay.

As the bAP is context specific (i.e., depends on the state of Na^+^ and K^+^ channels and the recent history of the membrane potential; see Bernard and Johnston, 2003), and may carry contextual information, we studied the adaptation of spike backpropagation delays to various stimuli, including suprathreshold stimulus currents that mimicked the pairing between the excitatory postsynaptic potential (EPSP) and somatic spike as occurs in the soma during STDP inducing protocols (Dan and Poo, 2004). The bAP delays correlate linearly with high frequency (>10 Hz) stimulation of membrane oscillations as seen *in vivo* (Bereshpolova et al., 2007). Although transient changes in the suprathreshold sinusoidal stimulus (SSS) frequency (<10 Hz) did not correlate with bAP delays, persistent injection of low frequency (7 Hz) suprathreshold sinusoidal current into the soma result in significant alterations of the bAP delay, suggesting an intrinsic adaptation of the bAP, which does not involve any synaptic modifications.

Some of the results have been published previously in abstract form (Buskila et al., 2013).

## MATERIALS AND METHODS

### ANIMALS

For this study, we used 2–5 weeks old Wister rats. All animals were healthy and handled with standard conditions of temperature, humidity, 12 h light/dark cycle, free access to food and water, and without any intended stress stimulations. All experiments were approved by the University of Western Sydney committee for animal use and care [Animal Care and Ethics Committee (ACEC) protocol #A9452].

### SLICE PREPARATION AND RECORDING

Wister rats were deeply anesthetized by inhalation of isoflurane (5%), decapitated, and their brains were quickly removed into ice-cold physiological solution (artificial cerebrospinal fluid, aCSF) containing (in mM): 125 NaCl, 2.5 KCl, 1 MgCl_2_, 1.25 NaH_2_PO_4_, 2 CaCl_2_, 25 NaHCO_3_, 25 dextrose and saturated with carbogen (95% O_2_–5% CO_2_ mixture; pH 7.4). Parasagittal brain slices (300 μm thick) encompassing the primary somatosensory cortex were cut with a vibrating microtome (Camden Instruments, UK) and transferred to a holding chamber containing carbogenated aCSF for 30 min at 35°C, which was then allowed to cool to room temperature for at least 1 h before recording.

### ELECTROPHYSIOLOGICAL RECORDINGS AND STIMULATION

The recording chamber was mounted on an Olympus BX-51 microscope equipped with IR/DIC (infrared/differential interference contrast) optics. During recordings, the slices were kept at room temperature, ~22°C, and constantly perfused (2–3 ml/min) with oxygenated solution as reported previously (Buskila and Amitai, 2010). Simultaneous whole-cell and cell-attached recordings were made from the soma and dendrites of neurons, respectively. Whole-cell recordings were performed from the soma of layer V pyramidal neurons in the somatosensory cortex with patch pipettes (5–7 Mømega) containing (in mM) 130 K-methansulfate, 10 HEPES, 0.05 EGTA, 7 KCl, 0.5 Na_2_GTP, 2 Na_2_ATP, 2 MgATP, 7 phosphocreatine, 0.1 Alexa Fluor-488 (Molecular Probes) and titrated with KOH to pH 7.2 (~285 mOsm). Cell-attached recordings from the apical dendrites were performed using patch pipettes (10–12 MΩ) filled with the same internal solution, excluding the fluorescent dye. The use of cell-attached patch recordings rules out the possibility that the findings are caused by washout of cytoplasmic constituents or capacitive load imposed on the cell by the patch pipettes. Stimulation protocols were designed using pClamp 10 software suit (Molecular Devices, Sunnyvale, CA, USA) and stimulation currents were injected through the recording electrodes. Voltages were recorded in current clamp mode using a multiclamp 700B dual patch-clamp amplifier (Axon Instruments, Foster City, CA, USA), digitally sampled at 30–50 kHz, filtered at 10 kHz, and analyzed off-line using pClamp software. The access resistance was corrected on-line and recordings were included in the analysis if the access resistance was <30 MΩ, and were considered stable and suitable for analysis if the access resistance, input resistance, and resting membrane potential did not change by more than 20% from their initial value during recording. At the termination of each experiment, the location and morphology of neurons were examined by fluorescence microscopy and digitally recorded (ROLERA-XR, Q-Imaging).

### DETERMINING THE RESONANCE FREQUENCY

In order to reveal the resonance frequency of the cells, we used the impedance analysis described by Gutfreund et al. (1995). In short, a 20-s subthreshold sinusoidal current with a linear increase in frequency from 0.1 to 20 Hz (chirp stimulation) was applied through the recording electrode. The impedance amplitude profile (ZAP) was generated by transforming the input current (*I*) and the voltage response (*V*) into the frequency domain using a fast Fourier transform (FFT), and then dividing the voltage transformation FFT(*V*) by the current stimulus transformation FFT(*I*). The stimulus file was generated by Python-based software, imported into pClamp, and applied as described above. The resonance frequency (*f*_R_) was determined as the peak of the ZAP profile.

### SUPRATHRESHOLD SINUSOIDAL STIMULUS PROTOCOL

**FIGURE 1:**
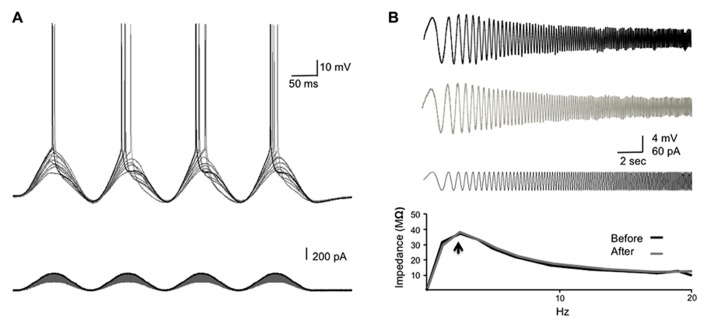
**Determining the resonance frequency and stimulus strength for SSS protocols.** **(A)** The experimental design. Increasing sinusoidal currents (10 pA steps) were injected into the soma until threshold was reached (Rh_sin_). The stimulus strength was set 20–40% above the Rh_sin_ to ensure a spike was elicited in >80% of the sine cycles. **(B)** A chirp stimulation was injected into the soma to reveal the resonance frequency (*f*_R_; black bottom trace). The membrane potential response to the chirp stimulus before and after the injection of the SSS protocol is shown (top and middle traces, respectively). The bottom graph shows the ZAP profiles as calculated from dividing the response FFT(*V*) by the stimulus FFT(*I*) revealing the resonance frequency (arrow), which did not differ following SSS protocols.

We wanted to introduce a persistent stimulus, which would mimic the pairing of the synaptic potential (EPSP) and the backpropagating spike in the soma, while avoiding actual synaptic plasticity that could complicate the interpretation of the results. We therefore designed a SSS protocol that elicited a single spike each cycle, coinciding with the rising phase of a sinusoidal current injected directly to the soma (**Figure 3A**). The stimulus protocol consists of 10 episodes of 10-s long suprathreshold sinusoidal current at 7 Hz with an inter-episode interval of 5 s, injected through the somatic recording electrode. To determine the stimulus strength, graded sinusoidal currents (10 pA increments) were injected into the soma until a single spike was initiated (sinusoidal rheobase – Rh_sin_; see **Figure 1**). To ensure spike generation in more than 80% of cycles, the stimulus strength was set to 120–140% of the Rh_sin_, ensuring a single spike at each cycle. The delay of the bAP from the soma to the dendrite was measured from the peak of the intracellularly recorded somatic AP to the peak of the bAP from cell-attached recordings in the dendrites.

### STATISTICAL ANALYSIS

Data is reported as mean ± SEM. Statistical comparisons were done using two-tailed unpaired Student’s *t*-test.

## RESULTS

We have studied the alterations in spike backpropagation delays in layer V pyramidal neurons of the somatosensory cortex. The average somatic resting membrane potential and spike amplitude were -62 ± 1 and 97.5 ± 4.6 mV (*n* = 9), respectively. Somatic input resistance and time constant were 120 ± 23 Mømega and 25 ± 4 ms respectively. The average propagation velocity of bAP evoked by step current (0.2–0.5 nA) was 0.68 ± 0.27 m/s (*n* = 9), consistent with previous reports on large layer V pyramidal neurons in the somatosensory and prefrontal cortex (Stuart et al., 1997a; Gulledge and Stuart, 2003; Bereshpolova et al., 2007). The average resonance frequency in the soma ranges between 1 and 3 Hz (average 2 ± 0.2 Hz; *n* = 9) and did not change significantly following the injection of suprathreshold sinusoidal current protocols (**Figure 1B**).

### bAP DELAY IS NOT SUSCEPTIBLE TO TRANSIENT STIMULI

**FIGURE 2:**
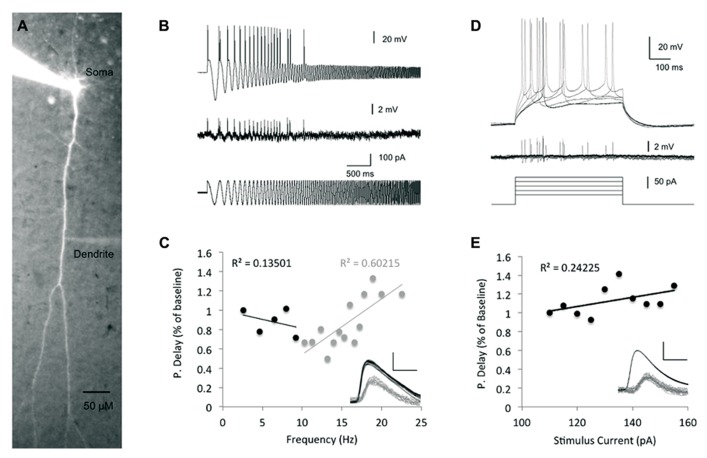
**The impact of stimulus intensity and frequency on spike backpropagation delay. (A)** Fluorescent image of a layer V pyramidal neuron following intracellular loading with Alexa Fluor-488, showing the cell morphology and placement of the recording electrodes at the soma and dendrite. **(B)** Propagation delay adaptation following alterations in the stimulus oscillation frequency. Sample traces [200 μm from the soma, baseline propagation velocity (PV) = 0.53 m/s] depict the experimental protocol. A sinusoidal chirp stimulation (bottom trace) was injected into the soma and the spike timing was recorded in both soma and dendrites (top and middle traces, respectively). **(C)** Scatter plot depicting the correlation between the bAP delay and the stimulus frequency (*n* = 5). Linear correlation was found only at frequencies >10 Hz (gray regression line). The propagation delay in the first cycle was used as the baseline. Inset – sample traces from **(B)** were superimposed to show the peak-to-peak delay (scale bar – vertical – 40 mV for somatic spikes in black and 2 mV for dendritic spikes in gray; horizontal – 2 ms). **(D)** Increasing suprathreshold current steps (500 ms, bottom traces) were injected into the soma through the recording electrode and recorded in both soma (top traces) and dendrites (middle traces). The first spike following the stimulus onset was used for comparison in **(E)**. The propagation delay at the lowest stimulus amplitude (110 pA) was used as baseline. **(E)** Scatter plot depicting the relationship between the injected current intensity and the bAP delay (*n* = 7). Line represents linear fit. Inset – sample traces of the bAP as recorded in the soma and dendrite (350 μm from the soma, baseline PV = 0.46 m/s) are superimposed to show the lack of significant change in each. Scale bar as in **(C)**.

To assess the bAP timing precision and adaptation to alterations in inputs from the cortical network, we measured the peak-to-peak bAP delay via simultaneous recordings from both soma (whole cell) and dendrites (cell attached, up to 500 μm from the soma). Previous studies reported on frequency dependence of the spike backpropagation (Spruston et al., 1995; Colbert et al., 1997; Gulledge and Stuart, 2003; Williams, 2004; Williams et al., 2007), in which persistent increase of firing frequencies (>10 Hz) correlated with higher attenuation of the bAP amplitude. On the other hand, a recent *in vivo* extracellular study showed that bAP invasion to the dendrites is highly reliable across a wide range of brain states, network activity and stimulus conditions and is only mildly modulated by neuronal firing frequency (Bereshpolova et al., 2007). To examine the dependence of spike backpropagation delay on transient changes in spike firing frequency, we injected a suprathreshold sinusoidal current with increasing frequencies from 0.1 to 100 Hz (chirp stimulation, **Figure 2B**) into the soma, which imitates a change in network input, such as the fluctuations in membrane potential (“up” and “down” states), in a more realistic fashion than the step current used previously (Waters and Helmchen, 2004). We then measured the bAP delay time and tested the correlation between the backpropagation delay and the stimulus frequency in which the spikes were initiated.

As seen in **Figure 2C**, at low frequencies (less than 10 Hz), there was a fairly weak correlation between the bAP delay and the stimulus frequency (Linear fit *R*^2^ = 0.13, *n* = 5), implying that the spike backpropagating delay is not modified by transient changes in the stimulus firing frequency, nor by slow membrane potential oscillations in that range. These results are aligned with a previous *in vivo* study, which found that firing frequencies below 10 Hz had only little effect on the extent of backpropagation (Bereshpolova et al., 2007). In contrast, during stimulus frequencies higher than 10 Hz, the bAP delay showed stronger correlation (Linear fit *R*^2^ = 0.6, *n* = 5) to the stimulus frequency as previously reported for both *in vivo* and *in vitro* studies (Spruston et al., 1995; Bereshpolova et al., 2007).

The mechanism underlying STDP involves the coincidence of bAP and EPSP. Previous studies showed that increase in synaptic current reaching the soma could increase the amplitude of bAPs (Sjöström et al., 2001; Stuart, 2001; Stuart and Häusser, 2001) and thereby impact LTP induction. Moreover, it has been shown that increased EPSPs reaching the soma did not affect the action potential amplitude in the soma, but increased its afterdepolarization, which could be associated with bAP amplification. Yet, it is not clear whether modifications of bAP amplitude were due to an increase in the current arriving to the soma *per se*, or caused by the impact of the increased EPSP along the dendrites and the processes associated with its increase (i.e., calcium influx). The amplitude of EPSP reaching the soma can be mimicked by simple inward current injections into the soma. To assess the impact of the stimulus strength on the spike backpropagation delay, we injected increasing step currents into the soma, which initiated action potentials with decreasing latencies from the stimulus onset as well as steeper increase of the membrane potential rising slopes and increasing afterdepolarizations (**Figure 2D**), and tested the correlation between the step stimulus strength and the backpropagation delay (**Figure 2E**). The linear regression line showed only a weak correlation between the stimulus strength and the backpropagation delay (*R*^2^ = 0.24), which is not surprising given the fact that the Na^+^ somatic spike has all or none characteristics.

### bAP VELOCITY DECREASES DURING SUPRATHRESHOLD SINUSOIDAL STIMULATION

**FIGURE 3:**
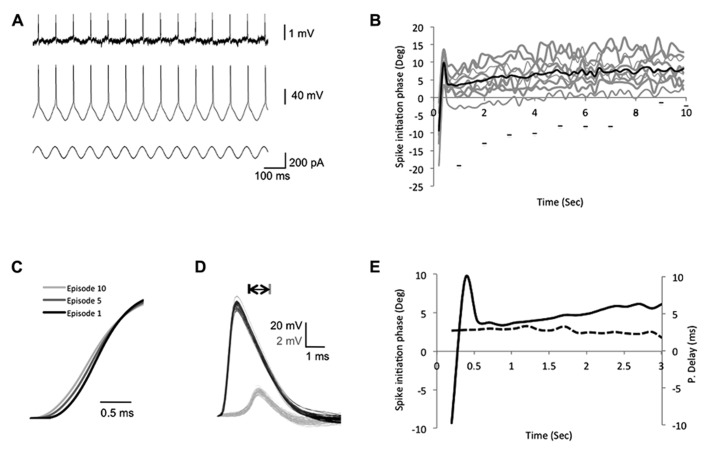
**Intrinsic plasticity inducing protocols modify the bAP initiation phase.** **(A)** The experimental design depicting the recording traces from both soma and dendrites during the suprathreshold sinusoidal stimulus protocol, which consists of 10 episodes. In each episode a 10-s suprathreshold sinusoidal current was injected into the soma (bottom trace) at 7 Hz and elicits spikes in the soma (middle trace), which paired with the peak of each rising cycle and propagated to the dendrites (top trace). **(B)** A plot depicting the somatic spike-timing phase during SSS protocol. Gray – single episodes, black – average. The horizontal bars represent the first spike in each episode (from left to right, *x*-axis represents episode number), to show the shift of the first spike across episodes. **(C)** Expansion of the sample average spike rising slopes of the 1st, 5th, and 10th episodes recorded in the soma depict a decrease of the rising slope across episodes. **(D)** Expansion of action potentials recorded in the soma (black) and apical dendrite (gray, 350 μm from the soma, PV = 0.41 m/s) during one episode. Note the consistent shape and amplitude of spikes during one episode. Scale bar – horizontal 1 ms; vertical – 20 mV black traces and 2 mV gray traces. **(E)** Expansion of the first 3 s in **(B)**. Solid line represents the average somatic spike-timing phase. Dashed line represents the average propagation delay time (values on the right ordinate in milliseconds), which did not correlate with the change in the spike initiation phase.

The above results indicate that under low frequency stimulation (<10 Hz), the bAP delay is robust and not affected by transient changes of either stimulus frequency or stimulus amplitude injected to the soma. However, previous studies showed that persistent stimuli, such as an increase in the firing frequency (Spruston et al., 1995; Colbert et al., 1997; Gulledge and Stuart, 2003; Williams, 2004; Williams et al., 2007) or LTP (Dan and Poo, 2004; Sjöström and Häusser, 2006; Williams et al., 2007), that modify the internal cellular environment (i.e., calcium influx and A-type K^+^ inactivation) can result in amplification or attenuation of the bAP amplitude. To study the adaptation of spike backpropagation delay following persistent constant changes in the input stimuli, we injected a suprathreshold sinusoidal current into the soma through the recording electrode, which elicited a single spike each cycle coinciding with the rising phase of the sinusoidal current (**Figure 3A**). To avoid bAP amplitude attenuation resulting from repetitive high firing rates (>10 Hz), or bAP amplitude amplifications due to cell resonance, we chose a stimulus frequency that did not result in the attenuation of the bAP amplitude, yet was different from the initial resonance frequency in the soma.

**FIGURE 4:**
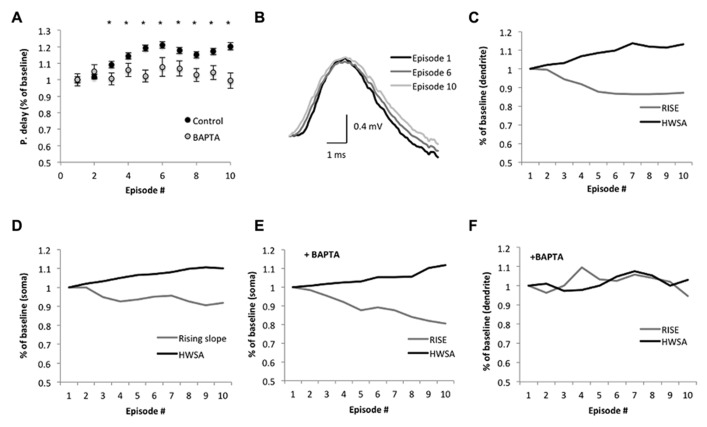
**Suprathreshold sinusoidal stimulus protocols modify spike backpropagation delays. (A)** Average propagation delay (shown as % of baseline) during the SSS protocol. Each dot represents the average delay in the episode. The average delay in the first episode was used as baseline. Significant alterations from baseline were noticed from the third episode onward (*p < 0.005, two-tailed Student’s *t*-test). The calcium chelator BAPTA abolished the significant increase in propagation delay in the last seven episodes, implying a calcium dependence (*n* = 4, *p* > 0.3; two-tailed Student’s *t*-test). **(B)** Average bAP traces in the dendrite (250 μm from the soma, aligned to the peak) at the 1st, 6th, and 10th episode show the changes in the slope. Scale bar (1 ms; 0.4 mV). **(C,F)** The average rising slope (10–90%) and HWSA of the bAP recorded in the dendrite during SSS show decreases and increases, respectively, across episodes as a % of baseline, which determined as the average rising slope in the first episode. **(D,E)** Modifications of the spike waveform across episodes plotted as a change from baseline. Black – HWSA, gray – 10–90% rising slope. **(F)** The addition of BAPTA to the recording electrode modified the significant alterations in the spike waveform.

During each episode, the spike initiation phase changed in a constant pattern, starting before the stimulus peak (off-phase) and reaching a steady state after 1 s (**Figures 3B,E**). Despite the modifications in the spike initiation phase, the spike shape remained stable in both soma and dendrites during individual episodes (**Figure 3D**). However, *across episodes*, a gradual decrease of the average action potential rising slope (10–90%) was detected in the soma, reaching a significant decrease of 6.3 ± 0.4% in the fifth and 8.9 ± 0.7% in the last episode (*n* = 6; *p* = 1.3 × 10^-^^17^ and 2 × 10^-^^17^, respectively, two-tailed Student’s *t*-test; **Figures 3C** and **4C**). Although previous studies indicated that the sinusoidal phase of the membrane potential at which the spike was initiated affected the bAP amplitude (Bernard and Johnston, 2003), the sensitivity to small alterations in phase was very minor (compare bAP amplitudes in Figure 2F in Bernard and Johnston, 2003), which is consistent with our results that show no correlation between the spike initiation phase and the propagation delay (**Figure 3E**).

The average backpropagation delay gradually increased across sinusoidal stimulus episodes, reaching a significant rise in the third episode. The delay increased by 9 ± 1% in the third episode and by 20 ± 2% in the 10th episodes (*n* = 6; *p* = 0.005 and 1.2 × 10^-^^12^, respectively, two-tailed Student’s *t*-test; **Figure 4A**), and was accompanied with a significant reduction of the rising slope (10–90%) of the bAP amplitude in dendrites (12 ± 1%, *n* = 6; *p* = 1.7 × 10^-^^11^, two-tailed Student’s *t*-test; **Figures 4B,C**) and soma (**Figure 4D**). The average bAP half-width spike amplitude (HWSA) in the dendrite was 2.39 ± 0.04 ms in the first episode and increased significantly (by 12 ± 1%) in the 10th episode (*p* = 0.04, two-tailed Student’s *t*-test). As the immediate effect of bAP is the increase in calcium concentration in the dendrites, and previous studies indicated that the intrinsic excitability in the dendrites is calcium dependent (Berridge, 1998), we tested the impact of calcium decrease on bAP during SSS protocol. The addition of the calcium chelator BAPTA (10 mM) into the recording electrode abolished the significant increase of the bAP delay across episodes (*n* = 4, *p* = 0.55, two-tailed Student’s *t*-test, comparing the 1st and 10th episode; **Figure 4A**), implying a calcium-dependent process in the change of bAP delay. As seen in **Figures 4E,F**, during the application of the SSS protocol in the presence of BAPTA, the average somatic HWSA increased by 11.7 ± 0.2% (*p* < 0.05, two-tailed *t*-test) and was also accompanied with a significant decrease of the rising slope (10–90%) by 17.3 ± 0.3% (*p* < 0.05, two-tailed *t*-test). However, calcium chelation by BAPTA had a profound impact on both HWSA and rising slope of the dendritic bAP, eliminating the significant modifications across episodes (compare **Figures 4C,F**).

## DISCUSSION

We have studied the adaptation of spike backpropagation delays in layer V pyramidal neurons of the somatosensory cortex during membrane oscillatory regimes.

Under our experimental conditions, the average propagation delay across episodes of SSS protocols increased in an accumulative fashion, until it reached steady state in the fifth episode (>300 spikes; **Figure 4A**). The phase of spike initiation within each episode changed in a unique pattern which was similar to the somatic action potential repolarization curve following a train of action potentials, described previously by Fleidervish et al. (1996). Thus we assume that the modifications in the spike initiation phase within each episode (**Figure 3B**) resembles spike frequency adaptation, which may be attributed to the fast inactivation of the Na^+^ current and positive shift of the threshold potential. We also observed a gradual modification of the first spike initiation phase of each episode within the SSS protocol (bars in **Figure 3B**), which might be attributed to the slow recovery of the Na^+^ channels from inactivation as reported previously (Fleidervish et al., 1996; Colbert et al., 1997). Yet, the alterations in the spike initiation phase during the episode did not affect the backpropagation velocity (**Figure 2E**). Moreover, at low frequencies (<10 Hz), transient alterations in the stimulus oscillation as well as modifications of the stimulus strength (**Figure 2**) showed only weak correlation to the backpropagation delay, implying that the bAP delay has robust features, which are not easily modified. These findings are aligned with previous *in vivo* study showing that at low frequencies the degree of bAP under varying conditions is quite uniform (Bereshpolova et al., 2007). However, during higher suprathreshold stimulus frequencies (10–25 Hz), the relationship between stimulus frequency and bAP delays showed higher correlation, which could be attributed to a calcium “dendritic switch” (reviewed by Sjöström et al., 2008), in which calcium influx into the apical dendrite is stronger with increasing network activity, hence boosting spike backpropagation. This further suggests that backpropagation can be modulated by the network state to play a role in cortical plasticity (Waters and Helmchen, 2004; Bereshpolova et al., 2007; Sjöström et al., 2008).

Alterations in the bAP delay could potentially be measured using two components. The first component can be detected through changes in the bAP waveform (i.e., decrease in the rising slope as seen in **Figures 4B,C** and widening of the HWSA), which are mainly due to modifications of the voltage-gated sodium current and Ca^2^^+^-dependent K^+^ channels. The second component is the propagation time along the dendrite, which can be measured by subtracting the changes in bAP rise time recorded in the dendrite from the total propagation and compare it to the original propagation delay. During the SSS protocol, the average rising slope of the bAP in the dendrite at the tenth episode decreases by 12 ± 1%, while the total increase in the propagation delay was 20 ± 2%. These results signify that under our experimental design, the alterations in the propagation delay include both components.

Migliore (1996) suggested two possibilities for spike attenuation and active propagation in the dendrites (see also Magee et al., 1998). The first is slow inactivation of the Na^+^ channels (due to the repetitive firing) which was experimentally reported (Colbert et al., 1997; Jung et al., 1997; Colbert and Johnston, 1998), and the other is through activation and inactivation of the calcium-dependent K^+^ channels (Jaffe et al., 1992; Bekkers, 2000), also called shunting conductance. Other factors that might impact the bAP propagation involves the increase in calcium concentration along the dendrite, which can boost the amplification of depolarizing events as well as voltage-dependent currents (such as *I*_h_) that are activated during the membrane oscillation.

### bAP DELAY IS AFFECTED BY CALCIUM-DEPENDENT PROCESSES

Various modulators may regulate spike backpropagation (Colbert et al., 1997; Astman et al., 1998; Hoffman and Johnston, 1998; Sjöström et al., 2008), yet, the principal factor governing spike initiation and propagation is the ratio between the Na^+^/K^+^ currents available at each location. The immediate effect of bAP is the increase in calcium entry into the dendrites through voltage-gated Ca^2^^+^ channels, which then affects Ca^2^^+^-dependent downstream processes such as Ca^2^^+^ -activated K^+^ conductance as well as slow inactivation of the Na^+^ conductance (Jaffe et al., 1992; Markram et al., 1997; Tsubokawa and Ross, 1997). That the gradual enhancement in propagation delay across episodes of the SSS protocol was due to a calcium-dependent process was shown by its ablation by the addition of the calcium chelator BAPTA to the recording electrode in the soma (**Figure 4A**). As calcium is involved in numerous processes that can influence dendritic excitability over a time period of minutes (Berridge, 1998), and it affects both rising slope and HWSA of the bAP in the dendrite (**Figure 4F**) we assume that multiple processes and not a single mechanism disrupt the adaptation of the bAP delay under BAPTA. These results are also aligned with a previous study by (Su et al., 2001), which suggests that a decrease in calcium concentration enhances intrinsic bursting via an increase of the persistent Na^+^ current. Hence, a reduction in calcium concentration upmodulates the persistent Na^+^ current by shifting its activation threshold (Li and Hatton, 1996), thus enhancing propagation velocity and reducing the delay.

The time course of the delay alterations were in the order of tens of seconds to minutes (**Figures 4A,B**), implying that the mechanism underlying this modification is probably mediated through a second messenger system, such as modulation of K^+^ channels by protein kinase A (PKA), or inactivation of Na^+^ channels by the Ca^2^^+^-dependent protein kinase C (PKC), as suggested by Hoffman and Johnston (1998). Activation of PKC has a dual impact on Na^+^ currents. On one hand it modulates the inactivation of Na^+^ channels, thus decreases their immediate availability, while on the other hand, on time scale of minutes, it shifts their voltage-dependent activation curve toward a more hyperpolarized potential (Astman et al., 1998), thus reducing spike threshold and changing both spike backpropagation amplitude and delay (Colbert and Johnston, 1998).

We therefore propose that following persistent repetitive invasion of bAPs to the dendrites, there is an increase in calcium influx, activation of Ca^2^^+^-dependent K^+^ currents (Bekkers, 2000) and fast inactivation of Na^+^ channels. These processes have fast kinetics, which alter the spike initiation phase in each episode as seen in **Figure 3D**. At later stages, the calcium influx leads to activation of PKC- and cAMP-dependent pathways as suggested previously (Astman et al., 1998; Colbert and Johnston, 1998; Hoffman and Johnston, 1998). These modifications further result in phosphorylation (and slow inactivation) of voltage-gated Na^+^ channels in both soma and dendrites. As dendritic and somatic Na^+^ currents recover with different time constants (5.6 and 4.1 s, respectively; see Colbert et al., 1997), the discrepancy between the dendritic recovery time to the somatic one shifts the Na^+^/K^+^ conductance ratio, which could explain the delay adaptation we are seeing across episodes. As the bAP is a regenerative process, a change in its voltage threshold due to different ratios of Na^+^/K^+^ conductance’s will result in a change of the propagation delay. Further investigation into the kinetics and range of the backpropagation delay adaptation following SSS will enhance our understanding of the underling processes.

## Conflict of Interest Statement

The authors declare that the research was conducted in the absence of any commercial or financial relationships that could be construed as a potential conflict of interest.

## AUTHOR CONTRIBUTIONS

Yossi Buskila, John W. Morley, Jonathan Tapson, and André van Schaik designed the approach; Yossi Buskila performed the measurements and analyzed the electrophysiological recordings. All authors were involved in writing the paper.
